# *Pucciniamodiolae* in North America: distribution and natural host range

**DOI:** 10.3897/mycokeys.39.27378

**Published:** 2018-09-11

**Authors:** M. Catherine Aime, Mehrdad Abbasi

**Affiliations:** 1 Purdue University, Department of Botany and Plant Pathology, West Lafayette, Indiana, USA Purdue University West Lafayette United States of America

**Keywords:** Neomycetes, Phytopathogens, Pucciniales, Uredinales

## Abstract

*Pucciniamodiolae*, a rust fungus pathogen of Carolina bristlemallow, *Modiolacaroliniana* (Malvaceae), is newly reported from North America, appears to be well established along the Gulf coast and is likely to have been introduced from South America. Its taxonomy, distribution and natural host range are discussed and a lectotype designated for this species. *Malvasylvestris* and *Alcearosea* are reported as new hosts for the rust. Additional new records for Malvaceae rusts are made for *P.modiolae* on *Alcearosea* from Brazil, *P.heterospora* on *Herissantiacrispa* in Florida and *P.heterogenea* on *Malva* sp. in Peru. Finally, an identification key for the microcyclic *Puccinia* species on members of Malvaceae in North America is provided.

## Introduction

Neomycetes are alien fungi entering a new area (country or continent), typically as a result of non-intentional human activity, that become established in the new region (Kreisel and Scholler 1994, Negrean and Anastasiu 2006). The most common origin for alien species of rust fungi in the USA appears to be South and Central America. In many cases, the pathogens are introduced concurrently with their host species, e.g. on crop plants, ornamentals or weeds.

*Pucciniamodiolae* P. Syd. & Syd. (Pucciniaceae, Pucciniales) is a microcyclic rust fungus that was originally reported on *Modiolaprostrata* A.St.-Hil. (=*M.caroliniana* (L.) G. Don; Malvaceae) from South America on the basis of specimens from Argentina and Uruguay (Sydow and Sydow 1904). *Modiolacaroliniana* is the only species in the genus *Modiola*, grows in disturbed vegetation and at forest margins and flowers in all seasons (Kearney 1951, Fryxell 1988). *Modiolacaroliniana* is believed to be native to northern Argentina and the Paraná basin of South America and probably came to the USA from southern South America in wool or cotton (Hanes 2015). Today, it is widely distributed as a weed in warmer parts of the world and is naturalised from the southern United States to northern Argentina including the West Indies. Despite the wide distribution of *M.caroliniana*, its parasitic rust, *P.modiolae*, has only been reported from Argentina and Uruguay (Lindquist 1982).

In this study, we examine numerous fresh collections and herbarium materials and conduct phylogenetic analyses of the 28S rDNA locus to provide the first reports of *P.modiolae* from North America, discuss its host range and distribution and establish a lectotype for this taxon. A key to the microcyclic *Puccinia* species on Malvaceae in North America is provided.

## Methods

Materials studied here were obtained from the Arthur Fungarium (PUR), the U.S. National Fungus Collections (BPI) and from fresh collections (listed in specimens examined below). Voucher specimens for new material are deposited in PUR. Rust spores and cross sections were routinely mounted in lactic acid in glycerol. Light microscopic analyses were performed using a Nikon Eclipse 80i microscope. Photomicrographs were obtained with a DS-Fi1 Nikon camera. In all studied specimens, thirty spores were randomly selected and measured.

DNA was extracted and the 5’ end of the nuclear 28S rDNA, amplified with rust-specific primers and sequenced following previous published protocols (Aime 2006, Aime et al. 2018). Sequences were edited using Sequencher 5.2.3 (Gene Codes Corp., Ann Arbor, MI) and aligned using the MUSCLE algorithm in Geneious 9.1.5 (Biomatters Ltd., Newark, NJ). Additional sequences of *Puccinia* species on Malvaceae were included for context from the studies of Aime (2006), Demers et al. (2015) and McTaggart et al. (2016). Phylogenies were reconstructed using maximum likelihood in RaxML v.2.2.3 via the CIPRES portal (Miller et al. 2010). Trees were visualised in FigTree v1.4.2 (http://tree.bio.ed.ac.uk/software/figtree/) and edited in Inkscape v2 (Free Software Foundation Inc., Boston, MA). Newly generated sequences are deposited in GenBank, accessions MH742974–MH743006.

## Results

**Figure 1.**
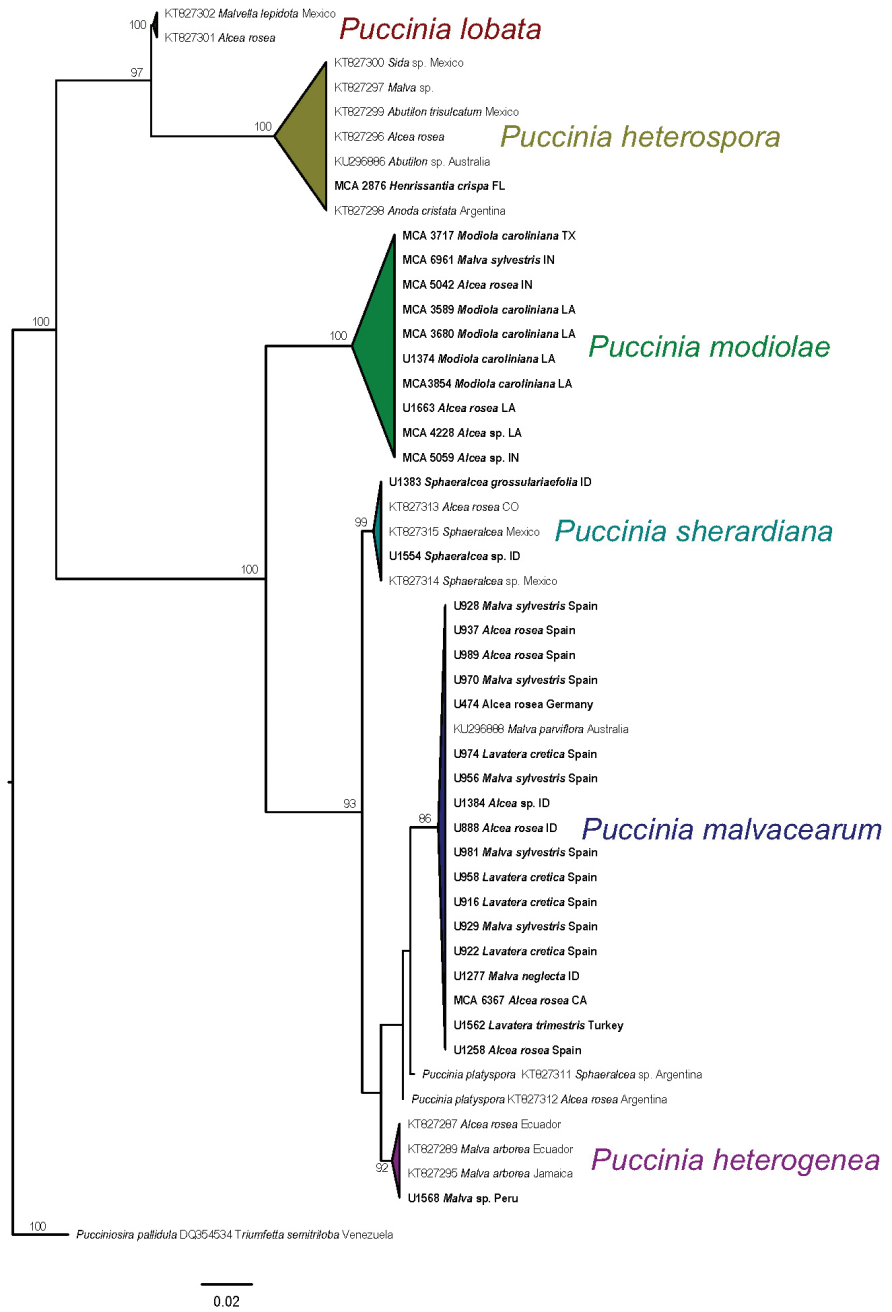
Maximum likelihood tree, based on 28S sequences, of *Puccinia* species on Malvaceae. Sequences newly generated for this study indicated in bold type. Numbers at nodes represent bootstrap support values. *Pucciniosirapallidula* was used as outgroup for rooting purposes.

Study of recently collected materials of malvaceous plants from Texas, Louisiana and Indiana revealed the widespread presence of *Pucciniamodiolae* along the Gulf coast on *Modiolacaroliniana* and occurring as far north as Indiana on new hosts *Alcearosea* L. and *Malvasylvestris* L. Examination of herbarium material also reveals *P.modiolae* as far south as Brazil on *A.rosea* (PUR N15322). Additional new records for Malvaceae rusts are made for *P.heterospora* on *Herissantiacrispa* in Florida and *P.heterogenea* on *Malva* sp. in Peru. In total, we generated 28S rDNA sequences for 32 collections of *Puccinia* species on Malvaceae, including ten collections of *P.modiolae* for phylogenetic analyses (Fig. 1); all sequences of *P.modiolae* shared 100% identity across the locus.

## Taxonomy

### Puccinia modiolae

Taxon classificationFungiORDOFAMILIA

P. Syd. & Syd., Monogr. Uredin. (Lipsiae) 1(3): 478 (1903) [1904]

**Figure 2.**
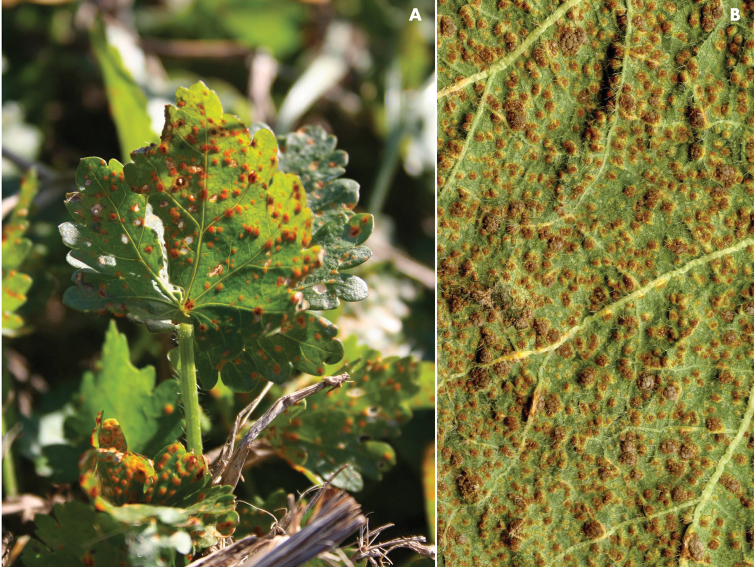
*Pucciniamodiolae*. **A** on *Modiolacaroliniana*, LA (MCA 3671) **B** on *Alcearosea*, IN (MCA 5059).

**Figure 3.**
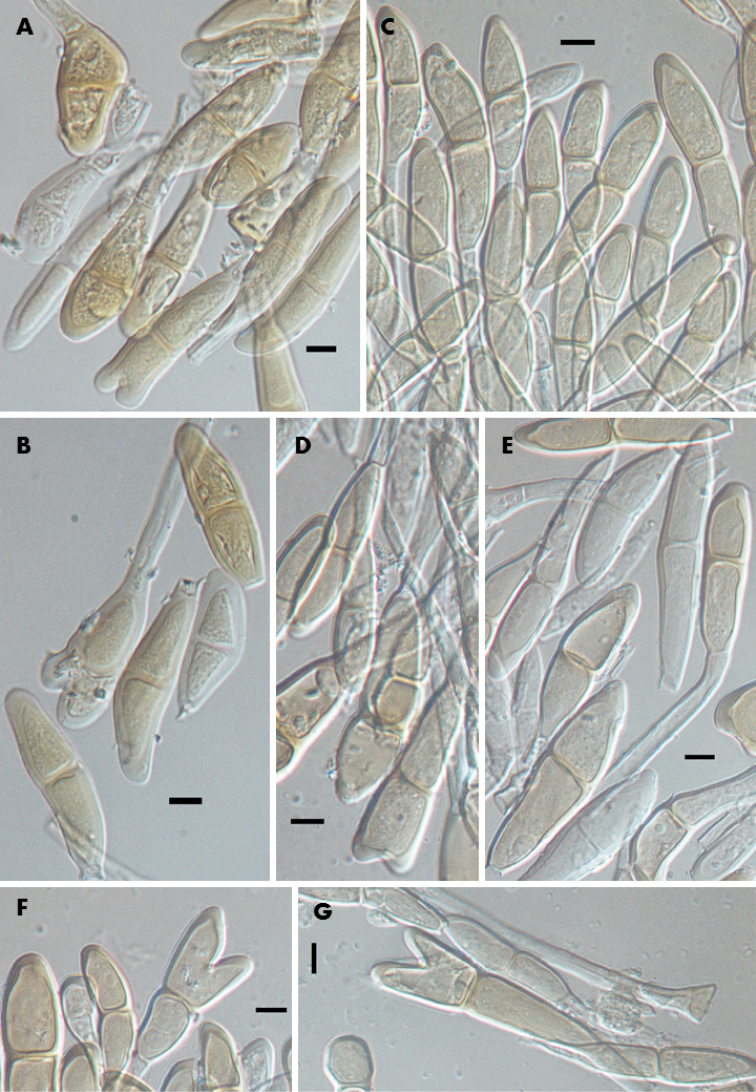
Teliospores of *Pucciniamodiolae*: **A–B** on *Modiolacaroliniana* (Lectotype PUR N6057) **C** on *M.caroliniana* (PUR N12041) **D** on *M.caroliniana* (PUR N12040) **E** on *M.caroliniana* (PUR N12550); **F** on *M.caroliniana* (PUR N12552) **G** on *Alcearosea* (PUR N12039). Scale bars: 10 µm.

**Figure 4.**
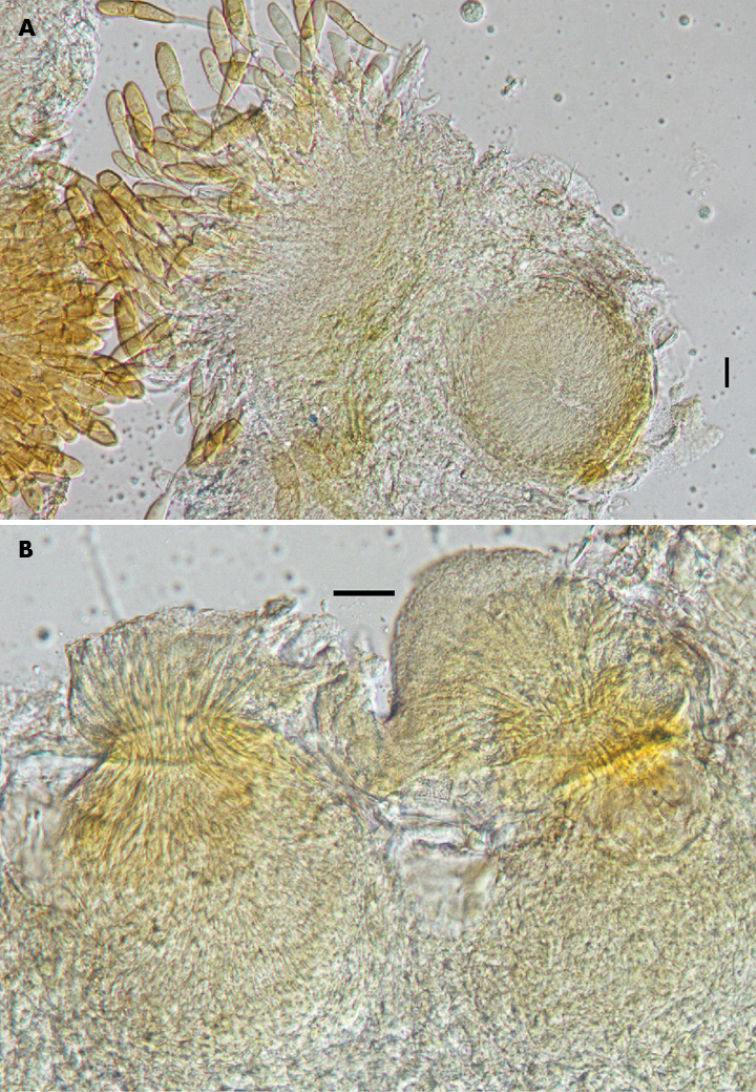
*Pucciniamodiolae* on *Modiolacaroliniana* (PUR N12551) **A** Spermogonium in connection with telium **B** Spermogonia with mass of spermatia on top. Scale bars: 25 µm.

P. malvacearum var. modiolae Pennington, Anales de la Sociedad Cientifica Argentina 55: 34 (1903). Figures 2–4. Syn.

#### Type:

Lectotype: on *Modiolacaroliniana* (as *M.prostrata*), Argentina, 1880–1881, C. Spegazzini, Decades Mycologiae Argentinae No. 10, PUR N6057, named as *P.malvacearum* (designated here). Isolectotype: BPI 086498.

#### Description.

Spermogonia usually epiphyllous, located on the opposite side of the telia in small groups, globose, 140–150 µm in diameter, yellowish-brown, with abundant and outward growing periphyses (Fig. 4). Telia mostly hypophyllous, occasionally on upper side of leaves and on petioles, round, compact, mostly in aggregated groups up to 3 mm in diameter, reddish-brown (Fig. 2). Teliospores diverse, with many anomalies because of the concretion of spores, mostly narrowly fusoid or linear, 31–81(–95) × 10.5–20 (–25) µm, attenuated above and below or notched at apex, not or hardly constricted at septum, wall smooth, hyaline to yellowish, 1.5–3 µm at sides, 3–8 µm at apex, pedicel hyaline, thick walled, persistent up to µm 150 µm (Fig. 3). One-celled and three-celled spores were rarely seen.

#### Specimens examined.

***Pucciniamodiolae*** – ARGENTINA: on *Modiolacaroliniana* (as *M.prostrata*), C. Spegazzini, Decades Mycologiae Argentinae No. 10, 1880–1881 (Lectotype, PUR N6057, as *P.malvacearum*; Isolectotype, BPI 086498, as *P.malvacearum*). USA: Indiana, Tippecanoe Co., Lafayette, *Alcearosea* L., M.C. Aime, MCA5059, 2012 Nov 05 (PUR N12038; GenBank accession #MH742985); *A.rosea*, M.C. Aime, MCA5042, 2012 Oct 01 (PUR N12039; GenBank accession #MH742978); West Lafayette, Purdue University Campus, *Malvasylvestris* L., Amnat Eamvijarn, MCA6961, 2016 Sept 16 (PUR N15171; GenBank accession #MH742977); Louisiana, East Baton Rouge Parish, Baton Rouge, Louisiana State University campus, *M.caroliniana* (L.) G. Don, Amnat Eamvijarn, U1374, July 2008 (PUR N12550; GenBank accession #MH742981); *M.caroliniana*, M.C. Aime, MCA3680, 2009 Mar 26 (PUR N12040; GenBank accession #MH742980); *M.caroliniana*, Don Ferrin, MCA3565, 2008 Mar 14 (PUR N12547, GenBank accession #MH742975); LSU Campus parking lot, *M.caroliniana*, Don Ferrin, MCA3589, 2008 May 14 (PUR N12552; GenBank accession #MH742979); Baton Rouge, private house, Malvaceae sp., Chris Clark, MCA4228, 2011 May 09 (PUR N22678; GenBank accession #MH742984); Bossier Parish, Red River Research Station, *M.caroliniana*, M.C. Aime, MCA4719, 2012 Apr 19 (PUR N12551); Evangeline Parish, Mamou, Main Street, Malvaceae sp., M.C. Aime, MCA3523, 2008 Feb 05 (PUR N22676); Tangipahoa Parish, 10 mi East of Independence, *M.caroliniana*, Charles Rush, MCA3854, 2009 Oct 22 (PUR N12549; GenBank accession #MH742982); St. James Parish, Convent, on the River Road in lawn next to Manresa House of Retreats, *M.caroliniana*, M.C. Aime & Tom Bruns, MCA3671, 2009 Jan 22 (PUR N12546); Orleans Parish, New Orleans, private residence, Malvaceae sp., Beth Kennedy, U1663, 2017 Mar 03 (PUR N22654; GenBank accession #MH742983); *Modiola* sp., M.C. Aime, MCA3568, 2008 Mar 23 (PUR N16658); Texas, Harris Co., Shell Station on Rt. 146, Seabrook Waterfront District, *M.caroliniana*, M.C. Aime, MCA3717, 2009 May 04 (PUR N12041; GenBank accession #MH742976). BRAZIL: Sao Paulo, *Alcearosea*, M. Figueiredo, J. Hennen s.n., 1999 Jan 12 (PUR N15322).

***Pucciniaheterogenea*** – PERU: Cajamarca Provence, Shudall, *Malva* sp., Jorge Diaz Valderrama, U1568, 2014 Dec 30 (PUR N12885; GenBank accession #MH743006).

***Pucciniaheterospora*** – USA: Florida, Monroe Co., Marathon, *Herissantiacrispa* (L.) Briz., M.C. Aime, MCA2876, 2004 Dec 31 (PUR N22677; GenBank accession #MH742974).

***Pucciniamalvacearum*** –USA: California, Alameda Co., Berkeley, *Alcearosea*, M.C. Aime, MCA6367, 2016 Aug 05 (PUR N15060; GenBank accession #MH743003); Idaho, Gem Co., *Alcearosea*, Krishna Mohan, U888, 2006 May 26 (BPI 878033; GenBank accession #MH742996); Canyon Co., Parma, *Alcea* sp., Ram Sampangi, U1384, April 2009 (PUR N16292; GenBank accession #MH742995); *Malvaneglecta*, Krishna Mohan, U1277, 2007 (PUR N16174; GenBank accession #MH743002); TURKEY: Bingöl Province, *Lavateratrimestris*, Lütfi Behçet, U1562, Jun 21 2014 (PUR N11582; GenBank accession #MH743004); SPAIN: Córdoba Province, near Montilla, *Malvasylvestris*, Walter J. Kaiser, U928, 2006 May 19 (BPI 878041; GenBank accession #MH742988); *M.sylvestris*, Walter J. Kaiser, U981, 2006 May 19 (BPI 878046; GenBank accession #MH742997); edge of wheat field, *M.sylvestris*, Walter J. Kaiser, U929, 2006 May 21 (BPI 878042; GenBank accession #MH743000); Cabra, edge of olive grove at Centro de Investigacion y Foirmacion Agraria, *M.sylvestris*, Walter J. Kaiser, U970, 2006 May 15 (BPI 878044; GenBank accession #MH742991); *M.sylvestris*, Walter J. Kaiser, U956, 2006 May 15 (BPI 878043; GenBank accession #MH742994); near Carcabury, *Alcea* sp., Walter J. Kaiser, U1258, April 2007 (PUR N16156; GenBank accession #MH743005); Córdoba, Colegio Mayor Universitario, Nuestra Senora de la Asuncion, Avenida Menendez Pidal, *Lavateracretica*, Walter J. Kaiser, U958, 2006 May 09 (BPI 878038; GenBank accession #MH742998); *L.cretica*, Walter J. Kaiser, U916, 2006 May 09 (BPI 878035; GenBank accession #MH742999); Malaga Province, outskirts of El Burgo, *Alcearosea*, U937, 2006 May 27 (BPI 875152; GenBank accession #MH742989); *A.rosea*, Walter J. Kaiser, U989, 2006 May 27 (BPI 878034; GenBank accession #MH742990); Jaén Province, Baéza, *L.cretica*, Walter J. Kaiser, U974, 2006 May 19 (BPI 878040; GenBank accession #MH742993); *L.cretica*, Walter J. Kaiser, U922, 2006 May 19 (BPI 878036; GenBank accession #MH743001); GERMANY, Thuringia, Weimar, *A.rosea*, G.R.W. Arnold, U474, 2004 Jun 22 (BPI 878032; GenBank accession #MH742992).

***Pucciniamalvastri*** –Arizona, Cochise, Cottonwood Canyon, Peloncillo Mountains, *Sphaeralcea* sp., George Cummins 61265, 1961 Sep 27 (topotype, PUR 59015).

***Pucciniasherardiana*** sensu Arthur (1922)–USA: Idaho, Canyon Co., Parma, *Sphaeralceagrossulariifolia* (Hook. & Arn.) Rydb., Ram Sampangi, U1383, April 2009 (PUR N12548; GenBank accession #MH742986); *S.grossulariifolia*, Krishna Mohan, U1554, 2009 Aug 18 (PUR N11663; GenBank accession #MH742987).

***Pucciniasphaeralceae*** –New Mexico, Mesilla Park, *Sphaeralceaangustifolia*, T. Cockerell 3478, 1896 Aug 01 (isotype, PUR 39636).

## Discussion

Phytoparasitic Neomycetes have the potential to cause great losses across the world via infestation of crops, ornamental plants and native flora (Scholler and Aime 2006). Introduction of alien phytoparasitic fungi also has ecological consequences which have been little investigated (Scholler 1999). There is no updated list of Neomycetes in the United States. However, alien rust fungi have had conspicuous economic and ecological consequences in North America. Here we report another introduced rust fungus, *P.modiolae*, as a new neomycete in the USA.

Pennington (1903) was the first to realise the difference between rust populations on *Modiola* compared to those on other members of the Malvaceae. He named the *Puccinia* species on *Modiola* as P.malvacearumvar.modiolae, based on material collected from Río Paraná, Argentina. Sydow and Sydow (1904) described the rust population on *Modiola* as a separate species based on different material (syntype) collected from Argentina and Uruguay, but designated no holotype for the species. They later considered P.malvacearumvar.modiolae as a synonym of *P.modiolae* in the appendix of their book (appendix to the first volume of Monographia Uredinearum, p. 892). Our phylogenetic analyses show *P.modiolae* and *P.malvacearum* are distinct species (Fig. 1); designation of a lectotype and isolectotype are made herein to stabilise the taxonomy for this species.

*Pucciniamodiolae* is a native rust fungus of South America and was most likely introduced in the USA by accompanying its host plant *Modiola*. The rust species is quite common on *Modiolacaroliniana* in Louisiana and was also found in Texas, making the Gulf coast a likely site for the original introduction of the rust species in North America. We are unable to pinpoint when *P.modiolae* was introduced into the USA. However, we were unable to locate any historical North American herbarium material of *P.modiolae* in BPI or PUR, nor were we able to find records of any rust species on *Modiola* in the USA, Canada or Mexico in all available literature, making it likely that *P.modiolae* became established in the southern USA probably no earlier than the second half of the 20^th^ century. Before the present study, *P.modiolae* was only known from Argentina and Uruguay. In Argentina, *Althaeaofficinalis* L., *Lavateraarborea* L. and *Malvaparviflora* L., in addition to *M.caroliniana*, have been reported as the natural host range of the rust species; only *M.caroliniana* is a reported host in Uruguay (Lindquist 1982). We have identified *Alcearosea* and *Malvasylvestris* as new hosts for this rust species, ranging from southern Brazil to the upper Midwest USA.

The presence or absence of spermogonia is one of the morphological features for distinguishing microcyclic rust fungi on Malvaceae members (Lindquist 1982). Our study revealed that this feature is stable and meaningful for separating *Puccinia* spp. on Malvaceae. All studied specimens of *P.modiolae* in this research produced spermogonia in close connection to telia (Fig. 4). Eight microcyclic *Puccinia* species have been reported on Malvaceae in North America thus far.

### Identification key to the microcyclic species of *Puccinia* on Malvaceae in North America

**Table:** 

1	spermogonia absent	**2**
–	spermogonia present	**6**
2	one-celled teliospores predominating	*** P. heterospora ***
–	one-celled teliospores rare or absent	**3**
3	telia usually dark brown	*** P. lobata ***
–	telia usually light brown	**4**
4	teliospore length mostly > 40 µm	*** P. malvacearum ***
–	teliospore length mostly < 40 µm	**5**
5	teliospore wall 2–3 µm thick at sides, much thicker above	*** P. anodae ***
–	teliospore wall 1–2 µm thick at sides, scarcely thicker above	*** P. exilis ***
6	teliospores with many anomalies because of the concretion of spores, making them appear notched at apex	*** P. modiolae ***
–	teliospores without spore anomalies	**(*P.sherardiana* s. lat.) 7**
7	teliospore length mostly > 50 µm, oblong-ellipsoid	*** P. sphaeralceae *** ^*^
–	teliospore length mostly < 50 µm, broadly ellipsoid	*** P. malvastri *** ^*^

## Supplementary Material

XML Treatment for Puccinia modiolae

## Footnotes

Arthur (1922) considered Pucciniamalvastri and P.sphaeralceae as synonyms of P.sherardiana Körn. However, P.sherardiana is an old world species reported originally from Armenia on Malvellasherardiana Jaub. & Spach. There are a few reports of this species in the old world from Central Asia (Ulyanishchen 1978) and Iran (Abbasi 2013). Determining whether P.malvastri and P.sphaeralceae are synonyms of P.sherardiana needs additional study including study of type materials and molecular analysis of old world material. However, study of the isotype of P.sphaeralceae (PUR 39636) and topotype of P.malvastri (PUR 59015) showed that these two species can be distinguished by distinct differences in size of teliospores (see the key), thus we retain them as separate species pending additional studies.
